# Personality and Perception: A Qualitative Investigation of Factors That Shape Mentorship Satisfaction Among Hand Surgery Fellows

**DOI:** 10.1016/j.jhsg.2025.100922

**Published:** 2026-01-22

**Authors:** Colby Newson, Steven Kozusko, Ava Chappell, Kacy J. Peek, A. Bobby Chhabra, Brent R. DeGeorge

**Affiliations:** ∗ Department of Plastic Surgery, University of Virginia Health System, Charlottesville, VA; † Department of Orthopedic Surgery, University of Virginia Health System, Charlottesville, VA

**Keywords:** Education, Hand surgery fellow, Mentorship, Qualitative analysis

## Abstract

**Purpose:**

Mentorship is critical to the professional and personal development of surgical trainees, influencing clinical competence and career advancement. Although mentorship during residency has been widely studied, little is known about mentorship experiences during hand surgery fellowship, a brief and high stakes training period requiring rapid relationship formation. This study explored mentorship experiences and satisfaction among hand surgery fellows and to assess how personality traits, demographics, and program factors influence mentorship quality.

**Methods:**

A mixed-methods study was conducted across 10 US hand surgery fellowship programs with Institutional Review Board approval. Participants completed an online questionnaire assessing demographics, fellowship characteristics, mentorship satisfaction, and personality traits. Semistructured interviews were conducted, transcribed, and analyzed using inductive content analysis to identify common themes. Quantitative data were analyzed using descriptive statistics, Pearson correlations, and analysis of variance with significance set at *P* < .05.

**Results:**

A total of 10 fellows from 10 programs participated. Fellows valued personal mentor attributes (eg, altruism, honesty, patience) over relational and professional qualities and rated shared demographics as least important. Mentorship satisfaction showed nonsignificant trends: higher satisfaction among fellows who rated themselves as more emotionally stable and lower satisfaction among those scoring higher in openness to experience. Program structure showed similar trends, with greater satisfaction reported by fellows with more mentors and fewer cofellows. Qualitative analysis identified seven themes: (1) ideal mentor qualities, (2) organic relationship building, (3) mutual investment and communication, (4) diverse mentor expertise, (5) demographics and representation, (6) sponsorship beyond mentorship, and (7) dealing with time and other challenges.

**Conclusions:**

Hand surgery fellows reported overall positive mentorship experiences and emphasized personal qualities over demographic similarity. Although quantitative associations were limited by sample size, trends suggest that personality traits and program structure may influence mentorship satisfaction. Recognizing these dynamics may help fellowship directors identify trainees at risk for poor mentorship alignment and implement early, targeted support within the 1-year fellowship’s limited timeframe.

**Type of study/level of evidence:**

Differential diagnosis/symptom prevalence study IV.

Mentorship is essential for the professional and personal development of surgical trainees, shaping their technical skills, decision-making abilities, and career trajectories. Strong mentorship has been linked to greater confidence in unsupervised performance and higher subjective skill levels among trainees.^1^ Beyond the clinical realm, high quality mentorship has also been shown to contribute to personal growth, well-being, and career longevity of trainees.^2^

Although much of the existing literature focuses on mentorship at earlier stages, such as medical school and residency, mentorship needs to evolve throughout training. Notably, there is a greater focus on technical training, job counseling, and professional societal involvement during later years of training.^3^ Despite its critical role in the transition to independent practice, mentorship during the fellowship stage, particularly in hand surgery, remains understudied. Although prior studies have explored challenges in mentorship through the lens of demographic disparities identified by trainees from underrepresented backgrounds, this study uniquely examines how personality characteristics may influence trainee success and satisfaction with mentor and mentee relationships. This question is particularly important in the 1-year hand surgery fellowship, where limited time makes the rapid formation of effective mentorship relationships essential for maximizing educational and professional development.

The purpose of this study was to better characterize mentorship experiences and satisfaction among hand surgery fellows using a mixed-methods/qualitative study design. Individual personality traits play a critical role in shaping the capacity to form effective mentorship relationships, and we will investigate how dispositional factors such as openness, agreeableness, and conscientiousness facilitate or hinder these interactions. Additionally, we examine whether personality traits, demographics, or program characteristics influence fellow satisfaction with mentorship. The data derived from this study may enable fellowship directors and program leaders to proactively identify fellows at risk for suboptimal mentorship relationships and to develop targeted interventions, such as mentor matching or communication training, that optimize educational experience, professional development, and well-being during fellowship.

## Materials and Methods

This study was approved by the Institutional Review Board at our institution. This study was performed according to the reporting guidelines of the consolidated criteria for reporting qualitative research (https://www.equator-network.org/reporting-guidelines/coreq/). Participants were recruited from 10 hand surgery fellowship programs across the country. Fellowship programs were selected by the senior author in collaboration with the Hand Fellowship Directors Association to ensure diversity in geographic region, primary training discipline, number of fellows, and faculty composition, with individual institutions omitted to preserve participant anonymity. Fellowship directors at these institutions were first provided with study details through email and subsequently forwarded the information to their fellowship cohort by email. Ultimately, 10 fellows—one from each institution contacted—chose to participate. Enrollment was intentionally limited to one fellow per program to preserve participant anonymity and to align with available funding and the time required to conduct in-depth qualitative interviews. A prestudy power analysis was not conducted. No subjects refused to participate in the study or dropped out. All questions, prompts, and guides were pilot tested by the senior authors and their institutional trainees; however, these data were not included in final analysis to avoid institutional bias.

All fellows were interviewed during the last quarter of their fellowship year (June to August). Participants first completed an anonymous online questionnaire, accessible using a unique, single-use link, provided in an email. The survey collected demographic information, fellowship characteristics, and personality traits using a 10-item measure of the Big Five Personality dimensions. The Five-Factor Model of personality, also known as the “Big Five,” conceptualizes human personality as comprising five broad and empirically derived dimensions—agreeableness, conscientiousness, extraversion, neuroticism, and openness—that collectively describe consistent patterns of emotion, cognition, and behavior across individuals and contexts.^4^ Respondents also rated the importance of various mentor attributes and their overall satisfaction with mentorship.

Following the questionnaire, participants took part in semistructured interviews designed to identify common themes in mentorship experiences. The semistructured interviews were performed using the Zoom online visual and audio recording platform, and full transcripts were collected with Zoom AI companion. The semistructured interviews were conducted by the first author who had been independently trained in qualitative study design and execution. All participants received compensation in accordance with institutional payment policies. Data were deidentified prior to analysis. Using a qualitative data analysis software with integrated artificial intelligence feature (Atlas.ti), data were coded line by line. Open codes were combined to form categories that were then combined into themes through abstraction using inductive content analysis. All data were verified by independent coding by two of the authors using a descriptive coding tree, and themes were derived directly from the data. Participants were not required to provide feedback on the findings. Participant quotations were presented to illustrate the themes.

Survey responses were analyzed using descriptive statistics. Pearson correlations and one-way analysis of variance (ANOVA) were used to explore associations between mentorship satisfaction and personality traits, demographics, and program characteristics. Given the small sample size (n = 10), statistical significance was set at *P* < .05, but findings were interpreted with emphasis on effect sizes and observed trends rather than significance alone. Partial eta squared (η^2^_p_) was calculated for ANOVA models, and 95% CIs for correlation coefficients were computed using Fisher z transformation.

## Results

Hand surgery fellows in this study represented diverse backgrounds. Half identified as women, 30% as non-White, 30% as immigrants or first-generation Americans, and 30% as the first in their family to pursue a career in medicine (Table 1). Fellows also trained in programs with varied structures, faculty compositions, and cohort sizes (Table 2).

**Table 1:** Demographic Characteristics of Interviewed Hand Surgery Fellows

Demographics		
Sex	Male	50%
	Female	50%
Average age (y)	34 ± 1.2	
Race		
	White	70%
	Non-White	30%
Immigrant or first-generation American		30%
First-generation in medicine		30%
Residency training program		
	Orthopedic surgery	60%
	Plastic surgery	40%

**Table 2:** Self-Reported Characteristics of Hand Surgery Fellowship Programs

Average number of faculty members (n)	9.8 ± 3.2	
Average number of hand surgery fellows	3.7 ± 1.5	
Educational model		
	Academic	7
	Priva-demic∗	3
Hand surgery training discipline	Orthopedic	4
	Combined – plastic surgery and orthopedic surgery	6

∗ A priva-demic fellowship was considered a hybrid model in which a community or private practice setting provides academic-style education and scholarly expectations.

**Figure 1:**
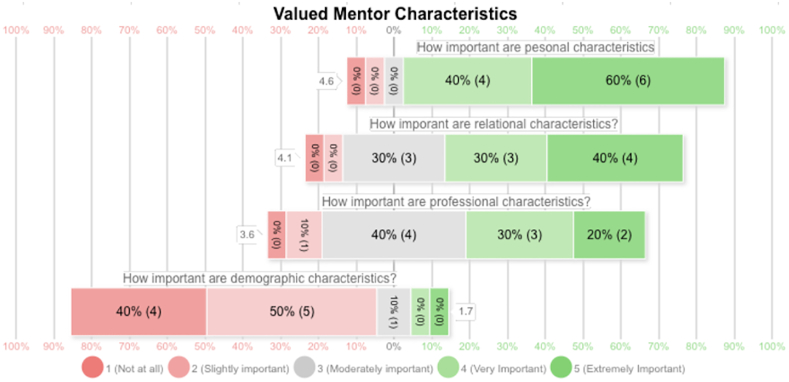
Likert scale comparison of ideal mentor attributes as reported by hand surgery fellows.

When ranking the most important mentor characteristics on a Likert scale, fellows placed highest value on personal attributes (eg, altruism, understanding, patience, honesty, motivation, and nonjudgmental attitudes), followed by relational attributes (eg, availability, accessibility, and dedication to the mentee relationship) (Fig. 1). Professional characteristics (eg, seniority, respect in the field, knowledge, and experience) ranked third in importance, whereas shared demographic characteristics were rated least important.

**Figure 2:**
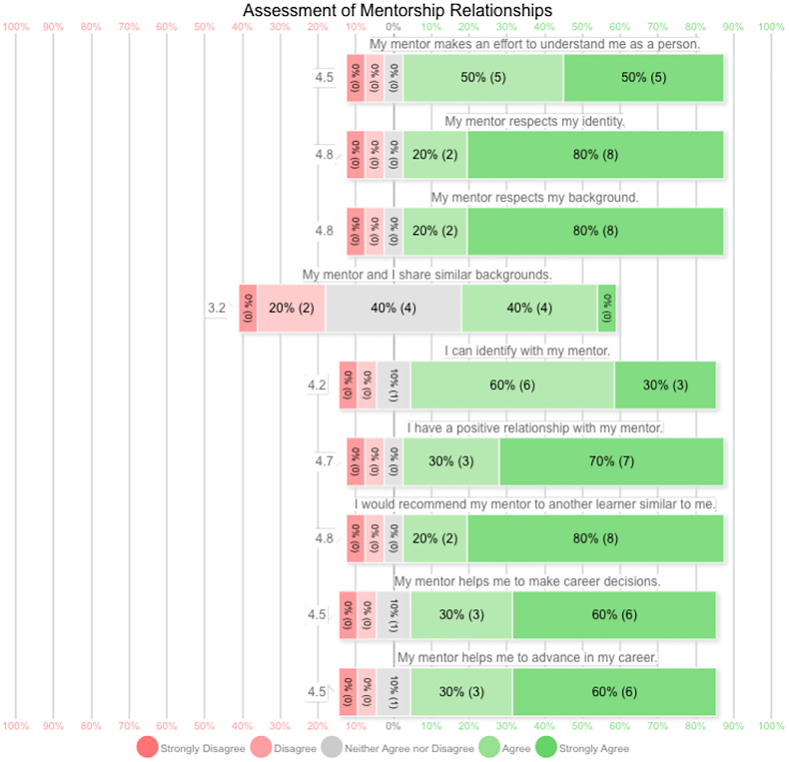
Likert scale assessment of different aspects of mentorship relationships as reported by hand surgery fellows.

Overall, fellows reported positive mentorship experiences and respect personally in their career development (Fig. 2). However, responses varied regarding if they believed a shared background with their mentors.

**Figure 3:**
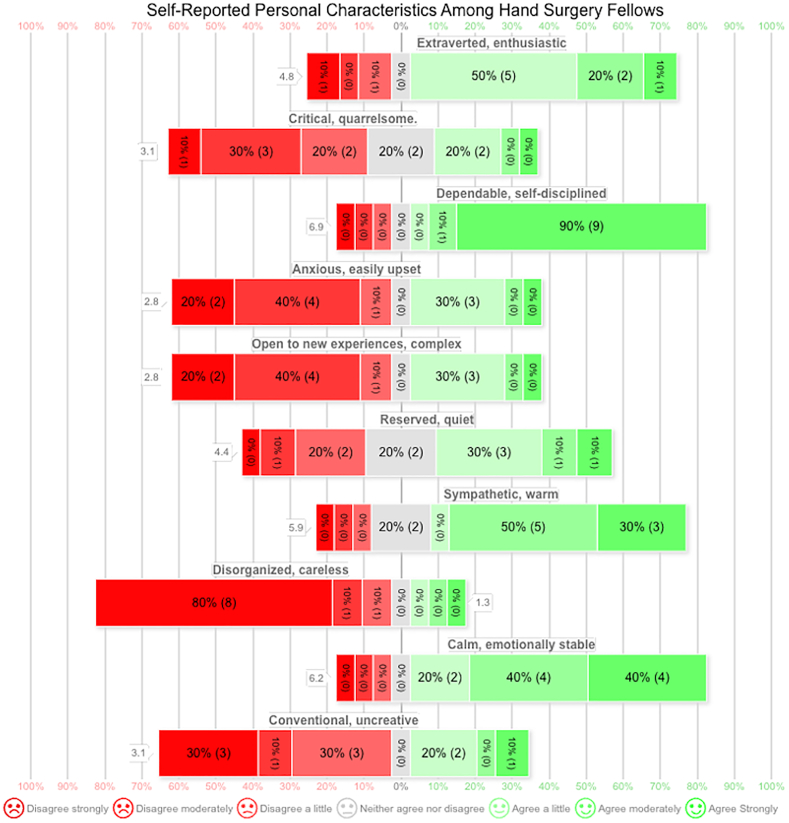
Likert scale distribution of self-reported personality characteristics among hand surgery fellows based on a 10-item measure of the Big Five Personality dimensions.

Fellows completed a 10-item measure of the Big Five Personality dimensions, rating responses on a 7-point Likert scale (Fig. 3). Pearson correlations were calculated between personality traits and satisfaction with the strongest mentorship relationship. Effect sizes were small for extraversion (r = –0.08), agreeableness (r = 0.19), and conscientiousness (r = 0.27). Emotional stability (r = 0.52) and openness (r = –0.59) showed larger effect estimates; however, confidence intervals were wide and none of the correlations reached statistical significance (all *P* > .05), indicating limited precision because of the small sample size (Table 3). These findings suggest possible trends rather than definitive associations.

**Table 3:** Pearson Correlation Coefficients Between Personality Dimensions and Mentorship Satisfaction (as rated on a scale of 0–100)

Personality Trait	Correlation with Mentorship Satisfaction (r)	Effect Size (r^2^)	95% CI for r	*P* Value
Extraverted	–0.08	0.01	–0.67 to 0.58	.834
Agreeableness	0.19	0.04	–0.49 to 0.72	.596
Conscientiousness	0.27	0.07	–0.44 to 0.77	.447
Emotionally stable	0.52	0.27	–0.16 to 0.84	.126
Open to experience	−0.59	0.35	–0.86 to 0.06	.071

Further analysis examined the relationship between program structure and mentorship satisfaction (Table 4). The number of faculty members showed no meaningful association with mentorship satisfaction (r = –0.33, *P* = .36), whereas the number of cofellows demonstrated a negative trend, with lower satisfaction reported in larger fellow cohorts (r = –0.62, *P* = .058) (Table 4). Although this relationship did not meet statistical significance, the effect size indicates a potentially important pattern that may warrant further study.

**Table 4:** Pearson Correlation Coefficients Between Numbers in the Program Structure and Mentorship Satisfaction (0–100 Scale)

Program Structure	Correlation with Mentorship Satisfaction (r)	Effect Size (r^2^)	95% CI for r	*P* Value
Number of faculty members	−0.33	0.11	–0.79 to 0.38	.358
Number of cofellows	−0.62	0.38	–0.90 to 0.02	.058

Analysis of variance assessing the relationship between the number of mentors and mentorship satisfaction showed a notable trend toward higher satisfaction among fellows with more mentors (F(2,7) = 3.97, *P* = .07). The corresponding effect size was large (partial η^2^ = 0.53), suggesting that the number of mentors may meaningfully influence satisfaction despite the lack of statistical significance (Table 5).

**Table 5:** ANOVA Comparing Reported Mentorship Satisfaction by Number of Self-identified Mentors

Number of Mentors	F statistic (DF)	Mean Satisfaction Rating ± SD	*P* Value	Partial η^2^
2	3.972 (2, 7)	72.5 ± 17.7	.070	0.53
3		93.3 ± 5.8		
5+		95.0 ± 8.7		

DF, degrees of freedom.

Finally, ANOVA indicated no significant differences in mentorship satisfaction based on sex or race (Table 6). In this study, sex was defined as the biological classification assigned at birth (ie male, female, or intersex), while gender was defined as a social construct encompassing an individual's identity and expression which may or may not correspond to sex assigned at birth. One-way ANOVA showed no significant differences in mentorship satisfaction by sex (F(1,8) = 0.99, *P* = .35, partial η^2^ = 0.11) or race (F(1,8) = 0.07, *P* = .80, partial η^2^ = 0.01). Effect sizes were small, indicating minimal variance explained by demographic factors.

**Table 6:** ANOVA Comparing Reported Mentorship Satisfaction by Sex and Race

Sex	F Statistic (DF)	Mean	*P* Value	Partial η^2^
Male	0.992 (1, 8)	86.0 ± 16.7	.348	0.11
Female		94.0 ± 6.5		
Race				
White	0.066 (1, 8)	89.3 ± 14.8	.803	0.01
Non-White		91.7 ± 7.6		

DF, degrees of freedom.

Analysis of semistructured interviews resulted in the development of seven overarching themes, with supporting quotations (Table 7). These themes included the following: (1) ideal mentor characteristics; (2) organic mentorship building; (3) need for mutual investment and communication; (4) value of mentors with diverse experiences and expertise; (5) role of demographics; (6) sponsorship as an extension of mentorship; and (7) challenges for mentees.

**Table 7:** Themes Identified From Semistructured Interviews With Representative Quotations

Theme 1: Ideal characteristics, including honesty, patience, and humility	“Humility is probably pretty important…it's easier for me to talk to someone about my struggles and my mistakes, if I also know they're willing to talk to me about theirs. So humility… as well as availability. I know I could call several people on the phone and they would answer immediately.” “Candidness is important. The willingness to tell you information that you don't want to hear, or that can be difficult to hear. People who, I think, have great emotional intelligence, people who understand interpersonal dynamics.” “I think someone who's responsive…someone who's like available and makes time for you is really nice. And then I think someone who's similar to you as a person. I think I have a mix. I have some mentors who are nothing like me as a person, and they offer their own strength.” “I think availability, honesty, dedication to their patients and their work. I think a certain degree of bluntness is important as well.”
Theme 2: Organic relationships and defining mentorship	“I never formally asked them to be my mentor. It was just kind of this natural evolution where I think we both recognize our roles and what we were doing, but never like labeled it.” “I may consider them a mentor, and I may get plenty out of that relationship without them ever feeling like I'm a Mentee. So no, I've never. I've never asked someone to define our mentee mentor relationship.” “I do think that just having questions that you want answered, and knowing who's in a position to answer them best is enough for me to know whether someone is a mentor, and I don't know that whether defining a mentor mentee relationship is as important because I can.”
Theme 3: Need for mutual investment and communication to sustain mentorship relationships	“I think communication is key, and not being a stranger, is probably the best way to maintain that mentee mentor relationship.” “Communication. And I think like not to overuse accountability, but on both sides. I think that as the mentee like, if you say you're going to do something, you need to do it…because every time someone in a position of power uses some of their credit to advance your career, you're kind of on their brand. If you do strong work with that, with that kind of gift, it's great. It kind of all pays forward.” “I think you can notice pretty quickly if someone's going to demonstrate follow-through after a meeting or two. That doesn't mean that I haven't held out reliance on mentors that, you know, may have not turned out to be the most invested. I think you probably need at least a couple meetings or interactions with the person to develop a relationship and kind of get a sense for what kind of mentor they'll be.”
Theme 4: Value of mentors with diverse experiences and areas of expertise	“I think their mentorship exists in silos in that way, or at least that's how I've organized it for the people here. And so, the positive memories, I think, are tied to their strongest attributes, and the things I want to take from them.” “But it's also good to have people who have all sorts of different training backgrounds and who are different levels of their career.” “I do think that I'm looking for mentors with different qualifications. Those qualifications include people who are technically skilled or whose clinical acumen I value I want to model when I am independently practicing.” “I think it's absolutely critical to have more than one mentor and I think they should offer different things to you.”
Theme 5: Role of demographics in mentorship	“I think again the thing you look for is do you have a relationship that you can kind of meld with? And can they provide to you what you're hoping to get out of that mentorship relationship whether that be research or life skills or sponsorship with other people in the society. I think the demographic shouldn't matter. To a certain extent, it's going to be more like your personal relationship, and how you meld with them.” “I don't think I put too much stock in the demographics of the mentors just by way of like who was available to me.”
Theme 6: Sponsorship as an extension of mentorship	“Mentorship is the person who you can ask questions about like, “Hey, at this case, what would you do?” and they just tell you what they would do, and go back to their lives; sponsorship is the person who texts you, and is like, “I have this great opportunity, and I thought would be perfect for it” and they’re kind of helping to push you forward and you can tell they're actively thinking about you rather than just thinking about you when you text them with a question.” “I really appreciate mentors who like every time they're in a situation where they could introduce you, or could try to help you in some way that they always do that, and they're always trying to think of how they can advance your learning, or advance your career, or your connections, or whatever it is.”
Theme 7: Challenges faced by mentees	“I think one of the challenging things, for me personally, is over committing and taking on more than you want, because you don't want to disappoint your mentors. That's where I've seen most of the struggle.” “I think the greatest challenge is just communication and that's setting up meetings. It's getting responses to emails or edits on projects or anything that requires them to find time in their schedule in a fairly rushed or time sensitive fashion, I believe it is difficult, not impossible, though just a personal challenge has been how to interact with mentors.” “From the mentees, probably just like fear of doing too much and being annoying, because you're like trying to balance this like, I want to be involved, but I don't want to annoy the attending that's super busy.” “I think it's lack of time. It takes effort and time to build a good relationship where you can build mutual trust.”

## Discussion

Hand surgery is a growing field, with an increasing number of accredited fellowship programs and a more diverse training population.^5^^,^^6^ Mentorship during the 1-year fellowship plays a crucial role in clinical development, professional identify, and well-being. However, mentorship is a dynamic process with evolving demands throughout training, and it remains unclear how well these needs are met during a 1-year fellowship. This mixed-methods study aimed to evaluate mentorship experiences during hand fellowship training and identify factors associated with higher mentee satisfaction.

Overall, mentees expressed satisfaction with mentorship in multiple domains including feeling understood, respected, and supported in career development. Quantitatively, no statistically significant associations were identified between mentorship satisfaction and personality traits, program features, sex, or race; however, several patterns emerged. Fellows who described themselves as more emotionally stable tend to report higher mentorship satisfaction, whereas those scoring higher in openness tend to report lower satisfaction. Similarly, program characteristics demonstrated meaningful trends: fellows with more mentors and those training in smaller cohorts generally reported higher satisfaction, and the effect size associated with number of mentors was large despite the lack of statistical significance. Taken together, these data suggest that mentorship satisfaction during hand surgery fellowship may be shaped by an interplay of interpersonal tendencies and program context, even if these relationships are not fully captured in a small sample.

Fellows consistently ranked personal and relational mentor qualities—such as honesty, humility, availability, and emotional intelligence—above demographic similarity. Given that orthopedic and plastic surgery—the primary feeder specialties for hand surgery—have historically been among the least diverse medical fields, mentorship diversity remains a challenge.^7^ In semistructured interviews, fellows emphasized the importance of building a network of mentors with varied experiences and expertise rather than focusing solely on demographic similarity. Many valued mentorships from individuals at different career stages and across various domains of professional development. Limited availability of sex and ethnically diverse mentors may partly explain this perspective. Prior research suggests that preferences for demographic concordance may vary by mentee identity, academic stage, and mentorship goals and may be more valued earlier in training.^8^ Specifically, sex-concordant mentorship may be particularly valuable for providing context and insight into shared experiences, conflict management, and career negotiation.^9^ Regardless of background, personal attributes that signal commitment, communication, and authenticity appear to have the greatest influence on mentorship quality.^10^

The role of personality in mentorship satisfaction remains unclear. It is uncertain whether specific personality traits inherently foster better mentorship relationships or if compatibility between mentor and mentee personalities plays a more notable role. However, understanding mentee personality traits could improve mentorship strategies. Previous work using the Hogan Personality Inventory found that orthopedic faculty rated individuals with characteristics such as procedural focus, rule compliance, sensitivity to patient needs, tactful communication, composure under pressure, intellectual curiosity, and adaptability as high performers.^11^ A better understanding of personality tendencies, particularly which are rated more critical to clinician performance, may allow mentors to provide more targeted and constructive feedback to trainees. A related study found that residents whose mentors had access to formal personality assessments reported higher mentorship satisfaction in some settings; however, effects were modest.^12^ Incorporating personality awareness into mentor training or mentor mentee matching may represent an opportunity for fellowship programs.

Effective mentorship relies on reciprocity, mutual respect, clear expectations, and shared values—key themes echoed in this study’s semistructured interviews.^13^ Fellowship programs vary in the structures in place to support these relationships. Previous research has shown that residents in programs with formal mentorship programs and those that met more frequently with mentors reported consisderably higher satisfaction than those in programs without such frameworks.^14^ Studies also suggest that mentees who select their own mentors tend to be more satisfied than those with assigned mentors.^15^ Beyond this initial “match”, a reciprocal loop—where both mentor and mentee invest time and maintain open communication—is crucial for sustaining an effective mentorship.^16^ Given our finding that greater numbers of cofellows trended to lower mentorship satisfaction, more formal mentorship programs may be particularly beneficial in larger cohorts to ensure adequate engagement.

Mentorship is a complex and multifaceted aspect of training. A key strength of this study is the integration of both qualitative and quantitative methods, which provided a broader range of responses and helped clarify certain themes that may not have been fully captured in multiple-choice survey responses. Additionally, participants came from diverse backgrounds and programs, offering unique perspectives.

This study is not without limitations. The small sample size limits generalizability and makes it difficult to draw definitive statistical conclusions. Future studies may expand to include a larger study population for quantitative analysis. Fellows also self-reported personality traits and did volunteer for study participation, which may introduce bias. The fellowship programs were solicited through known contacts of faculty at our institution, potentially introducing selection bias in the types of programs and participants represented. Because only mentee perspectives were collected, the findings capture a unidirectional view of the mentorship experience, despite mentorship being an inherently bidirectional process. Moreover, perceptions of mentorship may evolve over the course of the training and early career development. Future studies assessing both mentor and mentee perspectives at the beginning and end of fellowship, and even multiple years into practice, could provide valuable insight into how these relationships develop and whether mentorship dynamics change positively, negatively, or remain stable over time.

In summary, mentorship in hand surgery fellowship is a dynamic, interactive process that shapes professional growth within a brief training period. Although quantitative findings were limited by sample size, consistent trends paired with qualitative insights highlight the importance of personal and relational mentor qualities, the potential role of personality, and the added value of diverse mentor networks. Continued study of how personality and communication influence these relationships is essential, particularly as educators train a new generation of surgeons who face unique mental health and resilience challenges. Strengthening our understanding of mentorship will help future-proof the field of hand surgery.

## Conflicts of Interest

Given his role as Regional Editor for *The Journal of Hand Surgery Global Online*, Dr DeGeorge Jr. had no involvement in the peer review of this article and has no access to information regarding its peer review. Full responsibility for the editorial process for this article was delegated to Dr Fowler. No benefits in any form have been received or will be received by the other authors related directly to this article.
